# Electrophysiological Characterization of Networks and Single Cells in the Hippocampal Region of a Transgenic Rat Model of Alzheimer’s Disease

**DOI:** 10.1523/ENEURO.0448-17.2019

**Published:** 2019-02-22

**Authors:** Ingrid Heggland, Pål Kvello, Menno P. Witter

**Affiliations:** 1 Kavli Institute for Systems Neuroscience and Centre for Neural Computation, Egil and Pauline Braathen and Fred Kavli Centre for Cortical Microcircuits, Norwegian University of Science and Technology (NTNU), Trondheim NO-7491, Norway; 2 Liaison Committee between the Central Norway Regional Health Authority (RHA), the Norwegian University of Science and Technology (NTNU), Trondheim NO-7491, Norway; 3 Department of Teacher Education, Norwegian University of Science and Technology (NTNU), Trondheim NO-7491, Norway

**Keywords:** entorhinal cortex, fan cell, intracellular, neuronal excitability, stellate cell, voltage-sensitive dye imaging

## Abstract

The hippocampus and entorhinal cortex (EC) are areas affected early and severely in Alzheimer’s disease (AD), and this is associated with deficits in episodic memory. Amyloid-β (Aβ), the main protein found in amyloid plaques, can affect neuronal physiology and excitability, and several AD mouse models with memory impairments display aberrant network activity, including hyperexcitability and seizures. In this study, we investigated single cell physiology in EC and network activity in EC and dentate gyrus (DG) in the McGill-R-Thy1-APP transgenic rat model, using whole-cell patch clamp recordings and voltage-sensitive dye imaging (VSDI) in acute slices. In slices from transgenic animals up to 4 months of age, the majority of the principal neurons in Layer II of EC, fan cells and stellate cells, expressed intracellular Aβ (iAβ). Whereas the electrophysiological properties of fan cells were unaltered, stellate cells were more excitable in transgenic than in control rats. Stimulation in the DG resulted in comparable patterns in both groups at three and nine months, but at 12 months, the elicited responses in the transgenic group showed a significant preference for the enclosed blade, without any change in overall excitability. Only transient changes in the local network activity were seen in the medial EC (MEC). Although the observed changes in the McGill rat model are subtle, they are specific, pointing to a differential and selective involvement of specific parts of the hippocampal circuitry in Aβ pathology.

## Significance Statement

The hippocampal region, essential for episodic memory, is affected in the early stages of Alzheimer’s disease (AD). Here, we use the McGill-R-Thy1-APP transgenic rat model to study the effects of Amyloid-β (Aβ) pathology on networks and single cells in the hippocampal region. In young animals, we observed widespread intracellular Aβ (iAβ) accumulation, which later progressed to extracellular plaques. However, the *in vitro* physiology was largely unaltered, with only changes in single cell excitability of stellate cells in Layer II of MEC and network activation patterns in dentate gyrus (DG). Thus, these two components of the entorhinal-hippocampal network emerge as potentially more vulnerable in the context of Aβ pathology.

## Introduction

Alzheimer’s disease (AD), the most common cause of dementia, is a progressive neurodegenerative disorder. The neuropathological hallmarks include extracellular amyloid plaques and intracellular neurofibrillary tangles consisting of hyperphosphorylated tau, as well as cortical atrophy and cell loss. Areas affected by plaques and tangles in early stages of AD include the entorhinal cortex (EC) and the hippocampus (Braak and Braak, 1991; Thal et al., 2002). Neuron loss has been reported in subregions of the hippocampus (West et al., 1994; Simić et al., 1997; Price et al., 2001), and in particular Layer II of EC exhibits a substantial cell loss in patients in the early stages of AD as well as with mild cognitive impairment (Gómez-Isla et al., 1996; Kordower et al., 2001). The two main groups of principal neurons in Layer II, stellate cells in medial EC (MEC) and fan cells in lateral EC (LEC; Canto and Witter, 2012a,b), provide input to the hippocampus via the perforant path (Cappaert et al., 2015). In transgenic mice, it has been shown that both tau and amyloid-β (Aβ) pathology can spread through transsynaptic transmission, starting in EC (Harris et al., 2010; de Calignon et al., 2012), further implicating the entorhinal-hippocampal region in early stages of AD.

The original “amyloid cascade hypothesis” was formulated 25 years ago (Hardy and Higgins, 1992). Although the exact role of Aβ in the initiation and progression of AD is still highly debated, it is clear that Aβ is an important contributor to the pathologic processes (Herrup, 2015; Musiek and Holtzman, 2015). The research focus has shifted to include effects of soluble forms of Aβ (Haass and Selkoe, 2007) and Aβ peptide levels have been shown to have a higher correlation with cognitive decline than amyloid plaque load does (McLean et al., 1999; Näslund et al., 2000). Studies have shown toxic effects of Aβ oligomers on synaptic function and structure (Selkoe, 2008; Marchetti and Marie, 2011), which could lead to disruption of the normal neuronal function and subsequent aberrant network activity (Palop and Mucke, 2010a). Recent studies report changes in single neuron excitability in mouse models, including pyramidal cells of CA1 (Brown et al., 2011; Kerrigan et al., 2014), and frontal cortex (Kellner et al., 2014), as well as EC (Marcantoni et al., 2013; Xu et al., 2015) and dentate gyrus (DG; Hazra et al., 2013). Additionally, intracellular delivery of Aβ has been shown to increase neuronal excitability (Scala et al., 2015), and intracellular Aβ (iAβ) is found in EC and hippocampus of AD patients (Gouras et al., 2000; D'Andrea et al., 2002). As intracellular accumulation and cognitive deficits have been observed in animal models before formation of plaques (Billings et al., 2005; Leon et al., 2010), it is hypothesized that iAβ may play an important role in neuronal dysfunction in AD (Bayer and Wirths, 2010).

In this study, we use the McGill-R-Thy1-APP transgenic rat model which harbors human Aβ precursor protein (AβPP) with the Indiana and Swedish double mutations (Leon et al., 2010), and is one of the few rat models with a progressive plaque pathology. The first plaques appear in the subiculum at nine months of age and then spread to other parts of the hippocampus as well as EC (Heggland et al., 2015). A subtle cell loss (∼20%) has also been reported in the subiculum at 18 months (Heggland et al., 2015). By one week after birth, iAβ is observed (Leon et al., 2010), and Layer II of EC is one of the areas with initial high expression (Kobro-Flatmoen et al., 2016). From three months, the rats display cognitive impairments (Iulita et al., 2014) and metabolic alterations (Nilsen et al., 2014), and pre-plaque inflammation and changes in long-term potentiation have been described at later ages (Hanzel et al., 2014; Qi et al., 2014). In the present study, we investigated changes in excitability and activity patterns of the networks of the hippocampus and EC in acute slices. We used young pre-plaque animals, when only iAβ accumulation is present, as well as older animals, when plaques have started to appear. With the use of whole-cell patch clamp recording in acute slices from young animals, we investigated possible changes in the excitability of stellate and fan cells in Layer II of EC. We also assessed whether changes in electrophysiological properties at the network level were related to the developing pathology over time, with the use of voltage-sensitive dye imaging (VSDI).

## Materials and Methods

### Animals

All the experimental procedures were approved by the Local Animal Research Authority and followed the European Convention for the Protection of Vertebrate Animals used for Experimental and Other Scientific Purposes. The animals were kept on a 12/12 h light/dark cycle under standard laboratory conditions (19–22°C, 50–60% humidity) and had free access to food and water. A colony of transgenic McGill-R-Thy1-APP rats, based on two breeding pairs obtained from McGill University (Leon et al., 2010), was maintained at our university. The McGill-R-Thy1-APP rats carries the human *AβPP*_751_ including the Swedish double mutation and the Indiana mutation under the control of the *Thy*1.2 promoter. Quantitative PCR (qPCR) was used to decide the genotype of the transgenic rats (negative, homozygous or hemizygous for the transgene). Genomic DNA was isolated from samples of ear tissue with a High Pure PCR Template Preparation kit (11796828001, Roche Diagnostics). The transgene (human AβPP) and a normalization gene (GAPDH or β-actin) were detected using RT^2^ qPCR Primer Assays from QIAGEN (PPH05947A, PPR06557, and PPR06570C) with FastStart Universal SYBR Green Master (04913850001, Roche Diagnostics) on an Applied Biosystems StepOnePlus real-time PCR system (Life Technologies Ltd, Thermo Fisher Scientific). The ΔΔC_T_ values were calculated from the qPCR with a known homozygous sample as reference (Livak and Schmittgen, 2001).

### Slice preparation

For the VSDI, homozygous (+/+) transgenic rats and wild-type control animals (wt; WistarHan, Taconic) of both sexes at the ages of three, nine, and 12 months were used. For whole-cell patch clamp recordings, homozygous (+/+) transgenic rats and negative (–/–) littermates of both sexes at the ages of one and three to four months were included. The animals were anesthetized with isoflurane (IsoFlo vet., Abbott Laboratories), decapitated and the brain quickly removed from the skull and placed in ice-cold (0–4°C), oxygenated (95% O_2_/5% CO_2_) artificial CSF (ACSF) solution containing: 100 mM D-mannitol, 119 mM choline chloride, 2.5 mM KCl, 7 mM MgCl_2_, 0.5 mM CaCl_2_, 25 mM glucose, 1.25 mM NaH_2_PO_4_, 25 mM NaHCO_3_, 11.5 mM sodium ascorbate, and 3 mM sodium pyruvate. For rats of three months and older, a transcardial perfusion with ice-cold ACSF was done before decapitation, to remove blood and cool down the brain as quickly as possible.

For VSDI, the brain was cut in 400-µm-thick horizontal entorhinal-hippocampal slices on a vibratome (Vibratome 300 sectioning system, Vibratome). Slices ranged from approximate interaural levels 2.4–4.68 mm (Paxinos and Watson, 2007), containing mid to ventral levels of the hippocampus. The slices were placed on a membrane filter (JHWP01300, Omnipore membrane filter, PTFE, Merck Millipore) glued to a thin Plexiglas ring (11-mm inner diameter, 15-mm outer diameter) and held in a oxygenated moist interface chamber at 32°C for at least 1 h before transfer to the recording chamber. For holding and recording, the following ACSF was used: 126 mM NaCl, 3 mM KCl, 2 mM MgSO_4_, 2 mM CaCl_2_, 10 mM glucose, 1.2 mM NaH_2_PO_4_, and 26 mM NaHCO_3_.

For whole-cell patch clamp recordings, entorhinal slices of 400 µm were cut on a vibratome (Leica VT1000S, Leica Biosystems), either in the horizontal or semicoronal plane (20° angle with the vertical plane). Horizontal slices from middle dorsoventral levels, approximately interaural 2.9–4.4 mm (Paxinos and Watson, 2007), were used for recording MEC II cells, with the majority of the recorded cells found in the center of the mediolateral axis within each slice. Semicoronal slices were used for recording LEC II cells close to the rhinal fissure, at approximate rostrocaudal levels of 4.3–6 mm posterior to bregma (Paxinos and Watson, 2007). The slices were held in a submersion chamber with ACSF containing: 126 mM NaCl, 3 mM KCl, 3 mM MgCl_2_, 0.5 mM CaCl_2_, 10 mM glucose, 1.2 mM NaH_2_PO_4_, and 26 mM NaHCO_3_ at 37°C for 1 h, and then at room temperature until recording.

### VSDI

The slice was perfused with oxygenated ACSF at 34°C in a recording chamber mounted on a fluorescent microscope (Axio Examiner.D1, Carl Zeiss), and stained with the voltage-sensitive dye RH 795 (0.5 mg/ml ACSF; R-649, Invitrogen, Invitrogen, Life Technologies, Thermo Fisher Scientific) for 3 min, and the excess dye was washed out by perfusion of ASCF for 15 min before recording. The slice was illuminated from a halogen lamp (MHAB_150_W, Moritex) through a bandpass excitation filter (535 ± 25 nm) and a dichroic mirror (half reflectance wavelength of 580 nm), and the dye emission was passed through a longpass filter (50% transmittance at 590) and detected with a CMOS-camera (100 × 100 pixel array; MiCAM Ultima, Brainvision). For the three-month age group, a non-immersion Zeiss Fluar objective was used (NA = 0.25). For the nine- and 12-month groups, a water-immersion objective from Brainvision (NA = 0.35) was used, as we obtained this after the three-month group was recorded. A shutter (HL-151, Brainvision) controlled by the Brainvision acquisition software built into the light source was opened 500 ms before the start of the recording to reduce mechanical noise. The images were acquired at 1.0 ms/frame for 512 frames, and the first 50 frames were used to measure the optical baseline. An extracellular stimulation was applied after 50 ms with a tungsten bipolar electrode (tip separation of 150 µm) using either a single pulse with an amplitude of 0.2 or 0.6 mA of 300-µs duration, or four pulses at a frequency of 40 Hz with an amplitude of 0.2 mA. Eight recordings separated by 3 s were averaged to reduce noise. The stimulation electrode was placed in different areas of the hippocampal region: the border of the molecular and granule layer of the DG and Layers II/III of entorhinal. In the nine- and 12-month age group, the majority of the slices (38 of 44 slices) were also recorded with the GABA_A_ antagonist bicuculline added to the ACSF (5 µm; bicuculline methiodide; 14343, Sigma-Aldrich), to block the inhibition in the slice.

### Whole-cell patch clamp

All single cell recordings were performed at 34°C with perfusion of oxygenated ACSF containing: 126 mM NaCl, 3 mM KCl, 1.5 mM MgCl_2_, 1.6 mM CaCl_2_, 10 mM glucose, 1.2 mM NaH_2_PO_4_, and 26 mM NaHCO_3_. Principal cells in Layer II of MEC and LEC were identified using infrared differential interference contrast (IR-DIC) on an Axio Examiner.D1 microscope (Carl Zeiss) with a Zeiss Plan-Apochromat water dipping objective (20×; NA = 1.0), or an Olympus BX51WI microscope (Olympus) with an Olympus LC Plan FL objective (40×; NA = 0.8). Recording pipettes pulled from standard-walled borosilicate capillaries (3–8 MΩ pipette resistance; GC120F-10, Harvard Apparatus, Harvard Bioscience) were filled with intracellular solution containing: 120 mM K-gluconate, 10 mM KCl, 10 mM HEPES, 4 mM MgATP, 10 mM Na_2_-phosphocreatine, and 0.3 mM GTP. Biocytin (3–4%; B4261, Sigma-Aldrich) was added to the recording solution for later anatomic analysis of cell location and morphology. For a few of the cells (*n* = 34 cells), an Alexa Fluor hydrazide dye (405, 488, 468, or 633; Invitrogen, Invitrogen, Life Technologies, Thermo Fisher Scientific) was added to the intracellular solution instead of biocytin. Whole-cell recordings in current clamp mode were performed on two different setups. Recordings on the first setup was done with MultiClamp 700A and 700B amplifiers (Molecular Devices, Molecular Devices) in bridge mode and digitized with an InstruTECH ITC-1600 A/D interface (HEKA Elektronik) in combination with the acquisition software Chartmaster (HEKA). The second setup was equipped with a Multiclamp 700B amplifier and data were acquired with an InstruTECH ITC-18 board (HEKA) and the acquisition software Patchmaster (HEKA; RRID: SCR_000034). Recordings were made at sampling rates of 10, 25, or 50 kHz, depending on the length of the recording. Capacitance compensation was maximal and series resistance was compensated, and the seal resistance was above 1 GΩ. We did not correct for the liquid junction potential, which was calculated to be 15.8 mV.

To study general electrophysiological properties and firing frequencies of neurons, voltage responses to a series of 1-s-long current steps of 50 or 30 pA starting from – 300 pA were recorded. A protocol of 10-pA steps starting from 0 pA was used to measure the rheobase. In addition, we injected a sinusoidal current with a linearly increasing frequency (from 0 to 20 Hz) with a duration of 15 s, a so-called ZAP-protocol (Erchova et al., 2004), while recording the membrane voltage, and estimated the resonance frequency (frequency with largest amplitude response). In 11 cells, the resonance frequency could not be estimated, due to traces with noise or action potentials (APs).

### Histology

After recordings, the slices were fixed for minimum 24 h in 4% freshly depolymerized paraformaldehyde (w/v in 125 mM PB, pH 7.4) and then transferred to 20% glycerol and 2% dimethyl sulfoxide (DMSO) in 125 mM PB.

The slices from VSDI were subsequently cut at 50 µm on a freezing microtome (Microm HM430, Thermo Fischer Scientific). Half of the sections were mounted directly on Histobond^+^ slides and stained with cresyl violet to verify the regions of activity seen with the VSDI. After drying overnight on a heating plate (37°C) the sections were dehydrated in ethanol, cleared in xylene and rehydrated before staining with cresyl violet (1 g/l) for 10–15 min. The sections were then alternately dipped in ethanol-acetic acid (5-ml acetic acid in 1-l 70% ethanol) and rinsed with cold water until the desired differentiation was obtained, then dehydrated, cleared in xylene and coverslipped with Entellan (Merck KGaA).

The other half of the sections were stained with free-floating immunohistochemistry using the monoclonal anti-human Aβ antibody McSA1 (MM-0015-P; MédiMabs; RRID: AB_1807985), which is specific for human Aβ and stains both plaques and intracellular deposits (Grant et al., 2000; Leon et al., 2010). First, heat-induced epitope retrieval (HIER) was done at 60°C for 2 h in PB. After washing with PB (2 × 10 min) the tissue was permeabilized with 0.5% Triton X-100 in Tris-buffered saline (TBS-TX; 50 mM Tris and 150 mM NaCl; pH 8.0) for 10 min and blocked with 10% goat serum in TBS-TX for 30 min, before overnight incubation at 4°C with the primary antibody, McSA1 (1:4000). The following day, the sections were washed with TBS-TX (3 × 10 min) and incubated with a biotinylated goat anti-mouse secondary antibody (1:200, Sigma-Aldrich) for 90 min. After washing (TBS-TX; 3 × 10 min), incubation in ABC (PK-4000, Vectastain ABC kit, Vector Laboratories) for 90 min, washing with TBS-TX (3 × 10 min) and Tris-HCl (50 mM Tris adjusted to pH 7.6 with HCl; 2 × 5 min), and the sections were incubated in 0.67% diaminobenzidine (DAB) with 0.024% H_2_O_2_ in Tris-HCl for 30 min. After a final wash with Tris-HCl (2 × 5 min), the sections were mounted on Superfrost slides, dried overnight on heating plates, cleared with xylene and coverslipped with Entellan. A Zeiss Axio Imager.M1 microscope (Carl Zeiss) with a CX9000 camera (MBF Bioscience) was used to take brightfield photomicrographs of the sections, which were further processed with Adobe Photoshop CS6 (Adobe Systems; RRID:SCR_014199).

The slices from single-cell recordings were processed to visualize the morphology of the cells and to determine the intracellular expression of Aβ. HIER was applied at 60°C for 2 h in PB, and the slices were then washed 2 × 15 min in PB at room temperature followed by 5 × 15 min wash in 0.5% Triton X-100 in TBS-TX and incubation with the primary antibody, McSA1 (1:1000), at 4°C for 4 d. After rinsing 5 × 15 in TBS-TX, the slices were incubated overnight in room temperature with Alexa Fluor 488 conjugated to streptavidin (1:300; S11223) and a goat anti-mouse secondary antibody conjugated with Alexa Fluor 546 (1:200; A11003, Invitrogen). A subset of the slices was stained with the opposite combination of fluorophores (Alexa Fluor 546 streptavidin, S11225 and Alexa Fluor 488 goat anti-mouse, A11001). Subsequently, the sections were washed 3 × 15 min with TBS-TX, mounted, and coverslipped. The slices were scanned using a laser scanning confocal microscope (LSCM; LSM 510, Carl Zeiss) to determine the cell morphology and intracellular expression of Aβ. Alexa Fluor 488 was excited by an Argon/2 laser and the emission was registered through a 505- to 550-bandpass filter, whereas Alexa Fluor 546 was excited by a DPSS 461-10 laser and the emission was bandpass filtered at 575–615.

### Analysis of VSDI data

**Figure 1.**
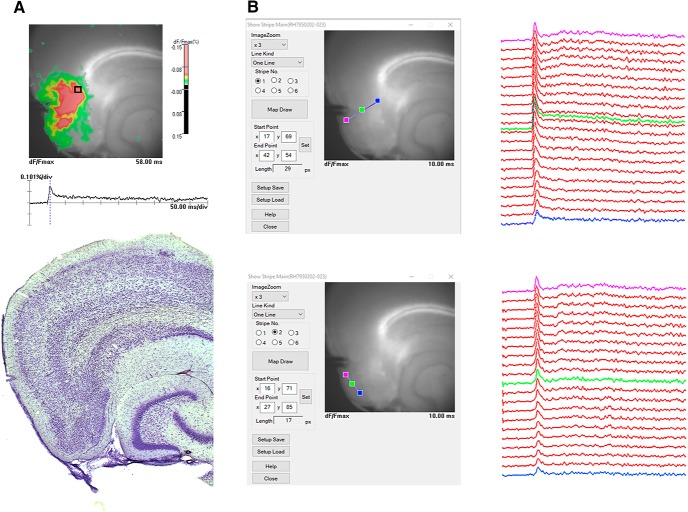
***A***, Example of VSDI imaging with stimulation in MECII in a horizontal slice (top) and the corresponding Nissl stain after histology (bottom). ***B***, Illustration of regions of interest (stripes) chosen to analyzed the VSDI signals in MEC, across layer (top left) and within the superficial layers (bottom left). To the right the corresponding traces for the voxels along the stripe is shown with the traces taken from the three color-coded voxels represented with the corresponding color.

The Brainvision analysis software (BV_Ana) was used to analyze the optical signals. Changes in membrane potential cause proportional changes in the emission of the voltage sensitive dye (Grinvald et al., 1988), and these were evaluated as fractional changes in the fluorescent signal (ΔF/F). All the optical signals were processed using spatial and cubic filters in BV_Ana. The first 50 frames were used as the average baseline, and the fractional optical signals were color-coded and superimposed on a brightfield image to represent the spread of neural activity in the slice (Fig. 1*A*). In the recordings from DG stimulation, we quantified the neural activation by calculating the integral (area under the curve) from optical traces in voxels in the molecular layer of DG and CA3, as this measure would represent the magnitude of the total membrane potential changes (Koganezawa et al., 2008). In addition, the total activated area in the whole slice after DG stimulation was quantified as the total number of pixels above threshold, with the threshold set to be 0.05% ΔF/F. The paired-pulse ratio (PPR) was calculated by dividing the maximal amplitude of second pulse by the first pulse, with a pulse interval of 25 ms. This was done for the voxels in each of the two blades of DG and an overall average PPR for DG was calculated for each slice. In recordings with stimulation in superficial MEC, stripes of voxels were analyzed. This was done both across layers and within the superficial layers in MEC, to evaluate the spread of the signal in the local network (Fig. 1*B*).

### Analysis of whole-cell patch clamp data

Analysis of the whole-cell current-clamp recordings was performed using the software Fitmaster (HEKA). The input resistance was calculated from the steady-state voltage response to injected current steps that did not elicit APs by fitting the quadratic equation:

$$△V\;=\;R_{N,0}△I\;+\;c_{AR}△I^{2}$$

Where *R_N,0_* is the voltage-independent input resistance and *c_AR_* is the coefficient of anomalous rectification (Waters and Helmchen, 2006). The membrane time constant, *τ*, was estimated by fitting a double exponential to the voltage response of a –300-pA current injection and using the higher value. The rebound potential was measured from a –300-pA current step (if the trace did not include a rebound AP), as the difference between the maximal value after the end of the stimulus (*V_max_*) and the baseline measured before the start of the stimulus (*V_baseline_*). The sag ratio was defined as:

$$Sag=(V_{baseline}-V_{ss})/(V_{baseline}-V_{\min})$$

where *V_min_* is the minimal value reached after the onset of the stimulus and versus*_s_*is the steady-state value of the voltage response to a –300-pA current step. The resting membrane potential (*V_m_*) was estimated by averaging a 10-s spontaneous recording. The following AP parameters were estimated from the first AP of the rheobase trace (the first trace in the rheobase protocol to elicit an AP): AP threshold (defined as maximum of the double derivative of the voltage response, found using a fit), AP amplitude (difference between maximum amplitude and AP threshold), AP half width (width at 50% of max AP amplitude), fast afterhyperpolarization potential (fAHP; minimum value directly after AP) and the depolarizing afterpotential (DAP; difference between the maximum value after the AP and the fAHP, in five cells with doublet spikes this could not be measured). The parameters fAHP and DAP were only measured in cells from MEC LII. We calculated AP amplitude, AP width at 0 mV and interspike interval (ISI) as a function of AP number from a positive current step (+200 or 210 pA), as well as the ratio between the first and the second ISI and the adaptation ratio (ISI_first_/ISI_last_). To look at the relationship between firing frequency and current, we measured the average firing frequency from current steps ranging from 200–500 pA. We also measured the instantaneous frequency between the two first APs (f_0_) and the two last APs (steady state, f_ss_). The AHP after the end of the current injection was also measured, for current steps ranging from 50–500 pA. The measures of firing frequencies and AHP as a function of current were only done on a subset of the cells, and for MEC this dataset only included cells from the one-month-old animals. Cells that had a V_m_ >–57 mV, AP amplitude <75 mV or a bridge balance >22 MΩ were excluded from the analysis, as well as putative interneurons.

The images from the LSCM were used to classify the neurons based on morphology. Cells in LEC LII that had a clear pyramidal (*n* = 9) or multiform (*n* = 17) morphology and cells in MEC LII with a clear pyramidal (*n* = 7) morphology, but not the intermediate cells types, were excluded from the analysis. Some cells were not filled well enough with biocytin to visualize the morphology (*n* = 9 for LEC and *n* = 5 for MEC). As the vast majority of the cells that were filled sufficiently were classified as fan cells (101 of 127 cells in LECII; 80%) or stellate cells (73 of 80 cells in MECII; 91%), we assumed that most of the non-filled cells would be of these types, and these were therefore included in the analysis. All the included cells from MEC displayed the known typical electrophysiological properties of stellate cells, including prominent sag and rebound.

### Statistical analysis

The quantitative VSDI data obtained in DG and MEC, was analyzed with respect to effect of genotype within each age group using a linear mixed model. Fixed factors were sex, genotype (+/+ or wild type) and where relevant, area (exposed and enclosed blade of DG) or distance from electrode in MEC, as well as the interaction between genotype and area/distance from electrode. A repeated effects variable with a diagonal or compound symmetry covariance structure (chosen based on convergence and information criteria) was included to account for several voxels (the regions of interest) being measured in each slice (intraslice variance).

A linear mixed model was used to estimate the effect of genotype on the measured electrophysiological parameters from the single cell recordings. Rat ID was added as a random effect to account for several cell recordings within one animal, and thus the values from each cell will not be independent. Genotype and age were included as fixed effects with two levels each (+/+ and –/–; one and three months). In addition, sex and experimental setup was included as a fixed effect to correct for possible differences that might bias the results. An extended model was also run to test for the possible interactions between genotype and age, and genotype and sex. On parameters with several measurements within the same cell (e.g., for several APs or current steps) the AP number or injected current was included as factors and as repeated measures with cell ID as the subject variable. The covariance structure for the repeated measures was compound symmetry or unstructured, based on which one had lower information criteria. The possible interaction between genotype and AP number or genotype and injected current was also included in the statistical model.

No corrections were done for multiple testing and results were considered statistically significant when *p* < 0.05. IBM SPSS Statistics, version 22 (IBM Corporation; RRID: SCR_002865) was used for the statistical analysis.

## Results

### No changes in fan cell physiology in LEC and subtle changes in stellate cell physiology in the MEC in homozygous McGill-R-Thy1-APP rats

To investigate whether basic electrophysiological properties or firing behavior were altered in the McGill-R-Thy1-APP transgenic rat, we performed whole-cell patch-clamp recordings in the current clamp mode of principal cells in Layer II of LEC and MEC in rats aged one and three to four months of age.

**Figure 2.**
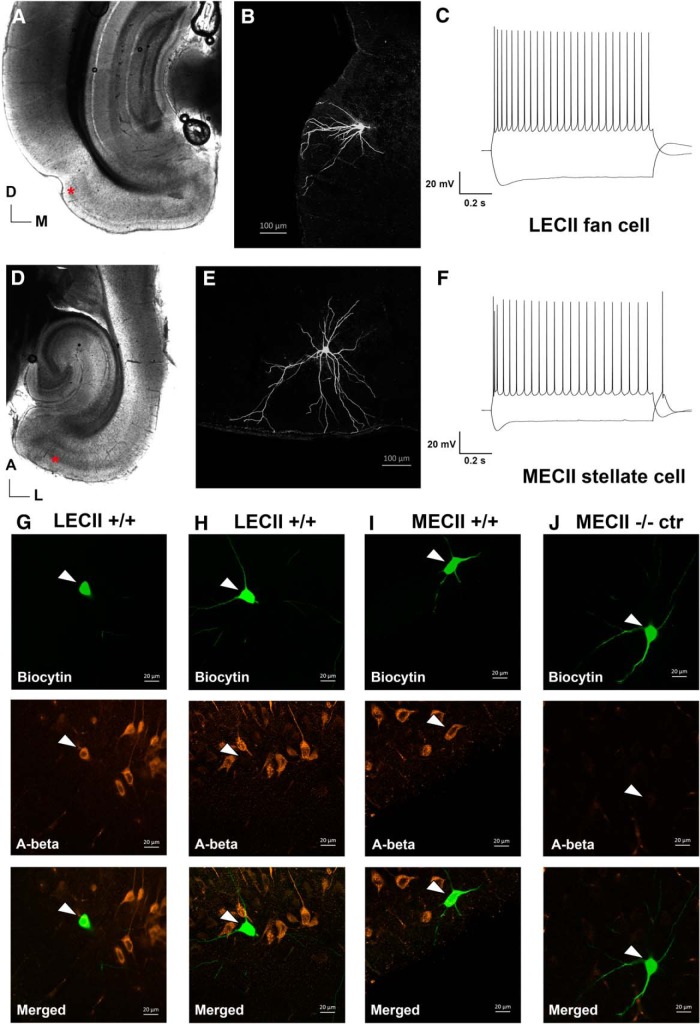
The characteristic morphology and electrophysiology of a fan cell and a stellate cell from EC Layer II and examples of intracellular expression of human Aβ in recorded cell. ***A***, Representative example of a semicoronal slice used for recording cells in LEC. The asterisk ventral to the rhinal fissure marks the location of the patched cell shown in ***B***. ***B***, Confocal image showing the morphology of a typical fan cell in Layer II of LEC, close to the rhinal fissure (location marked in ***A***). ***C***, Trace from the fan cell in ***B*** showing voltage responses to hyperpolarizing and depolarizing current steps of ±300 pA. ***D***, Representative example of a horizontal slice used for recording cells in the MEC. The asterisk marks the location of the cell shown in ***E***. ***E***, Confocal image showing the morphology of a typical stellate cell in Layer II if MEC (location marked in ***D***). ***F***, Trace from the stellate cell in ***E*** showing voltage responses to hyperpolarizing and depolarizing current steps of ±300 pA. LEC, lateral EC; MEC, medial EC. ***G***, Confocal scan from a three-month-old homozygous McGill-R-Thy1-APP transgenic rat showing a fan cell in LEC LII with iAβ. ***H***, Confocal scan from a one-month-old homozygous transgenic rat showing a stellate cell in MEC LII with iAβ. ***I***, Confocal scan from a one-month-old homozygous transgenic rat showing a fan cell in LEC LII without expression of iAβ. ***J***, Confocal scan from a one-month-old negative control animal showing no expression of human iAβ. The cells were filled with biocytin during recording and visualized with streptavidin labeled with Alexa Fluor 488. The presence of the anti-human Aβ antibody McSA1 was visualized with Alexa Fluor 546. LEC, lateral EC; MEC, medial EC; A, anterior; D, dorsal; L, lateral; M, medial.

We included 111 fan cells from LEC in the electrophysiological analysis (*n* = 47 cells from transgenic animals and *n* = 64 cells from control animals; aged one and three to four months). Since iAβ reportedly aggregates preferentially in LEC close to the rhinal fissure (Kobro-Flatmoen et al., 2016), we selectively recorded fan cells in LEC superficially in Layer II and just ventral to the rhinal fissure, in semicoronal slices (Fig. 2*A*). Most of them displayed the typical morphology, with apical dendrites fanning out toward the pial surface and only a few or no basal dendrites (Tahvildari and Alonso, 2005; Canto and Witter, 2012a; Fig. 2*B*). Fan cells showed a low sag and rebound potential, no spike doublets/triplets or DAP, but had a relatively high input resistance and time constant (Fig. 2*C*; Canto and Witter, 2012a,b). The majority of the fan cells recorded in homozygous (+/+) transgenic rats (89%; 31 of 35 neurons) stained positive for iAβ (Fig. 2*G*). A few neurons in the +/+ animals were not Aβ immunoreactive (four of 35 fan cells; example in Fig. 2*H*), whereas in negative littermates (–/–) none of the neurons showed immunoreactivity to human Aβ.

None of the measured basic electrophysiological or AP parameters of fan cells differed between the transgenic and control animals (Table 1; Extended Data Table 1-1). Similarly, there was no significant effect of genotype on the measured wave form parameters and firing properties (Table 2; Extended Data Table 2-1).

**Table 1.** Results from the mixed linear model analysis for electrophysiological parameters of LEC II fan cells in homozygous transgenic animals (+/+) and controls (–/–)

		Estimated marginal means		*p* values for test of fixed effects		
		–/–	+/+	Genotype	Age	Sex
a	Input resistance (MΩ)	138.0	129.8	0.299	0.085	**0.013** 1
b	Time constant, τ (ms)	28.9	30.7	0.230	0.746	**0.001** ^2^
c	Sag ratio	0.803	0.799	0.776	0.284	0.890
d	Rebound (mV)	5.45	5.51	0.926	0.534	0.666
e	V_m_ (mV)	–69.3	–70.7	0.126	**0.022** ^3^	0.204
f	Rheobase (pA)	71	72	0.934	**0.034** ^4^	**0.006** ^5^
g	AP threshold (mV)	–45.0	–46.8	0.054	0.313	0.119
h	AP amplitude (mV)	91.8	91.7	0.898	0.563	0.098
i	AP half width (ms)	1.071	1.128	0.152	0.665	0.659

Basic electrophysiological and AP properties from all individual cells are represented in Extended Data Table 1-1; *n* = 111 cells from 40 animals. V_m_, resting membrane potential. Values printed in bold face identify significance.

^1^Effect size: 21.9 MΩ (males higher). ^2^Effect size: 5.6 ms (males higher). ^3^Effect size: –2.2 mV. ^4^Effect size: 18.9 pA. ^5^Effect size: 24.5 pA (males lower).

**Table 2.** Estimated *p* values for AP parameters as a function of AP number and firing properties of LEC LII fan cells

		Genotype	Age	Sex	AP no.	AP × genotype
a	AP amplitude	0.855	**0.002** 1	**0.024**^2^	**0.000**	0.059
b	AP width	0.388	0.195	0.620	**0.000**	0.123
c	ISI	0.166	0.124	**0.039**^3^	**0.000**	0.317
d	ISI1/ISI2	0.698	**0.007**^4^	**0.006**^5^	-	-
e	Adaptation ratio	0.957	0.237	**0.014**^6^	-	-
		Genotype	Age	Sex	Current	Current × genotype
f	Average frequency, *f*	0.554	0.634	0.577	**0.000**	0.425
g	*f_0_*	0.936	0.334	0.122	**0.000**	0.157
h	*f_ss_*	0.666	0.650	0.578	**0.000**	0.977
i	AHP	0.774	0.659	0.434	**0.000**	0.656

AP and firing properties of LEC LII fan cells in homozygous transgenic rats (+/+) and control animals (–/–) are shown in Extended Data Table 2-1. Values are estimated *p* values from the mixed linear model, *n* = 111 cells from 40 animals; for firing frequencies and AHP *n* = 67 cells from 26 animals. ISI, interspike interval; adaptation ratio, first ISI/last ISI; *f_0_*, instantaneous firing frequency between two first APs; *f_ss_*, instantaneous firing frequency between two last APs. Values printed in bold face identify significance.

^1^Effect size: 2.5 mV. ^2^Effect size: 1.7 mV (males lower). ^3^Effect size: 9.8 ms (males higher). ^4^Effect size: –0.07. ^5^Effect size: 0.07 (male higher). ^6^Effect size: 0.08 (male higher).

Extended Data Table 1-1 Basic electrophysiological and AP properties of fan cells in LEC LII in homozygous transgenic rats (+/+) and negative control animals (–/–) at one and three months of age. ***A***, Input resistance measured from a series of current steps. The membrane time constant, τ (***B***); sag ratio (***C***); and rebound potential (***D***) all measured from a current step of –300 pA. ***E***, Resting membrane potential, V_m_. ***F***, Rheobase, measured by current steps increasing by 10 pA/step. AP threshold (***G***), AP amplitude (***H***), and AP half width (***I***) all measured from the current step at rheobase. Values from all individual cells are shown (*n* = 111 cells in 40 animals). Download Extended Data Table 1-1, TIF file.

Extended Data Table 2-1 AP and firing properties of LEC LII fan cells in homozygous transgenic rats (+/+) and control animals (–/–), in both age groups. ***A***, AP amplitude as a function of AP number. ***B***, AP width at 0 mV as function of AP number. ***C***, ISI, interspike interval as a function of spike interval number. ***D***, Ratio of the two first interspike intervals (ISI1/ISI2) and adaptation ratio (first ISI/last ISI). Values in ***A–D*** are measured from a +200- or 210-pA current (*n* = 111 cells in 40 animals). Average firing frequency, *f* (***E***); instantaneous firing frequency between two first spikes, *f_0_* (***F***); instantaneous firing frequency between two last spikes, *f_ss_* (***G***); afterhyperpolarizing potential after end of current step (***H***), all plotted as a function of current (*n* = 67 cells in 26 animals). All values are shown as estimated marginal means and SEs from the mixed linear model. Download Extended Data Table 2-1, DOCX file.

In total, 78 stellate cells in Layer II of MEC were included in the analysis (*n* = 38 cells from homozygous transgenic rats and *n* = 40 cells from negative littermates, aged one and three to four months). The stellate cells, recorded in horizontal slices, were mainly located superficially in Layer II (Fig. 2*D*), and not at extremes of the mediolateral axis (i.e., not close to the border to parasubiculum or LEC). The majority of the stellate cells displayed the typical morphology, with dendrites radiating from the soma (Fig. 2*E*), though some cells had intermediate stellate to pyramidal morphologies (Canto and Witter, 2012b; Fuchs et al., 2016). All included cells showed a prominent sag (low sag ratio) and rebound potential, and rebound spikes after a hyperpolarizing pulse were not uncommon (Fig. 2*F*). In addition spike doublets or triplets could be seen in the start of spiking trains, and a fAHP and DAP was clearly seen after single APs (Canto and Witter, 2012b). When staining for iAβ, 96% of the recorded stellate cells in slices from transgenic were Aβ-immunoreactive (25 of 26 cells; example in Fig. 2*I*). No Aβ-immunoreactive neurons were observed in the control slices (Fig. 2*J*).

Of all the electrophysiological properties measured in stellate cells, two parameters were altered in transgenic compared to control rats (Tables 3, 4; Extended Data Tables 3-1, 4-1). The fAHP displayed a slight but significantly increased hyperpolarization in the homozygous +/+ rats compared to the controls (Table 3, row k), and f_0_ was also significantly increased in the +/+ transgenic rats (Table 4, row g; estimated effect 36.2 Hz).

**Table 3.** Estimated marginal means and *p* values for electrophysiological parameters of MEC II stellate cells

		Estimated marginal means		*p* values for test of fixed effects		
		–/–	+/+	Genotype	Age	Sex
a	Input resistance (MΩ)	50.3	55.5	0.327	0.762	0.133
b	Time constant, τ (ms)	13.3	13.3	0.945	0.121	**0.004** 1
c	Sag ratio	0.580	0.607	0.094	0.230	**0.034** ^2^
d	Rebound (mV)	7.6	7.3	0.711	0.135	0.849
e	V_m_ (mV)	–64.7	–64.7	0.937	0.217	0.229
f	Resonance frequency (Hz)	4.5	4.5	0.982	**0.025** ^3^	0.069
g	Rheobase (pA)	123	111	0.375	0.690	0.225
h	AP threshold (mV)	–47.9	–48.9	0.229	0.148	0.246
i	AP amplitude (mV)	89.1	86.4	0.105	0.231	0.974
j	AP half width (ms)	0.991	0.980	0.820	0.155	0.856
k	fAHP (mV)	–51.6	–53.1	**0.034**	**0.000** ^4^	0.161
l	DAP (mV)	1.9	2.3	0.479	0.125	0.752

Basic electrophysiological properties of MEC LII stellate cells in homozygous transgenic rats (+/+) and negative control animals (–/–) for all cells are provided in Extended Data Table 3-1. Values are estimated marginal means and *p* values from the mixed linear model, *n* = 78 cells from 30 animals. V_m_, resting membrane potential. Values printed in bold face identify significance.

^1^Effect size: 2.2 ms (males lower). ^2^Effect size: 0.026 (males higher). ^3^Effect size: –0.65 Hz. ^4^Effect size: –4.0 mV.

**Table 4.** Estimated *p* values for AP parameters as a function of AP number and firing properties of MEC LII stellate cells

		Genotype	Age	Sex	AP no.	AP × genotype
a	AP amplitude	0.318	0.463	0.812	**0.000**	0.258
b	AP width	0.264	0.670	0.246	**0.000**	0.589
c	ISI	0.576	0.593	0.977	**0.000**	0.685
d	ISI1/ISI2	0.449	0.657	0.416	-	-
e	Adaptation ratio	0.818	0.737	0.862	-	-
		Genotype	Age	Sex	Current	Current × genotype
f	Average frequency, *f*	0.611	-	0.829	**0.000**	0.884
g	*f_0_*	**0.042**	-	0.332	**0.000**	0.884
h	*f_ss_*	0.839	-	0.870	**0.000**	0.984
i	AHP	0.561	-	0.629	**0.000**	0.922

Values are estimated *p* values from the mixed linear model, *n* = 78 cells from 30 animals; for firing frequencies and AHP *n* = 38 cells from 16 animals. ISI, interspike interval; adaptation ratio, first ISI/last ISI; *f_0_*, instantaneous firing frequency between two first APs; *f_ss_*, instantaneous firing frequency between two last APs. Values printed in bold face identify significance.

Extended Data Table 3-1 Basic electrophysiological properties of MEC LII stellate cells in homozygous transgenic rats (+/+) and negative control animals (–/–) at one and three months of age. ***A***, Input resistance measured from a series of current steps. The membrane time constant, τ (***B***); sag ratio (***C***); and rebound potential (***D***), all measured from a current step of –300 pA. ***E***, Resting membrane potential, V_m_. ***F***, Membrane resonance frequency in response to a ZAP current. ***G***, Rheobase, measured by current steps increasing by 10 pA/step. AP threshold (***H***), AP amplitude (***I***), AP half width (***J***), fAHP, fAHP (***K***), and DAP (***L***), all measured from the first AP of the current step at rheobase. Values from all individual cells are shown (*n* = 78 cells in 30 animals). Download Extended Data Table 3-1, TIF file.

Extended Data Table 4-1 AP and firing properties of MEC LII stellate cells in homozygous transgenic rats (+/+) and control animals (–/–), for both age groups in ***A–D***, one-month group in ***E–H***. ***A***, AP amplitude as a function of AP number. ***B***, AP width at 0 mV as function of AP number. ***C***, ISI, interspike interval as a function of spike interval number. ***D***, Ratio of the two first interspike intervals (ISI1/ISI2) and adaptation ratio (first ISI/last ISI). Values in ***A–D*** are measured from a +200-pA current step (*n* = 78 cells in 30 animals). Average firing frequency, *f* (***E***); instantaneous firing frequency between two first spikes, *f_0_* (***F***); instantaneous firing frequency between two last spikes, *f_ss_* (***G***); and afterhyperpolarizing potential after end of current step (***H***), all plotted as a function of current (*n* = 38 cells in 16 animals). All values are shown as estimated marginal means and SEs from the mixed linear model. Download Extended Data T, TIF file.

In the statistical model, age and sex were included as fixed effects, and on several of the electrophysiological parameters these had significant effects (Tables 1–3). In this study, the aim was to investigate the effects of genotype, but these results for age and sex underline the importance of including these as factors in the statistical analyses.

### VSDI and Aβ immunoreactivity of the hippocampal region

**Figure 3.**
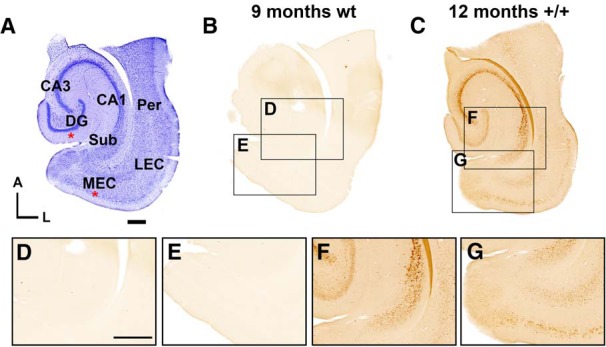
Horizontal slices from control animals and homozygous McGill-R-Thy1-APP rats were used for VSDI. ***A***, Example of a horizontal slice stained with cresyl violet showing the different areas targeted for stimulation (red asterisks). ***B***, Example slice from a nine-month-old wild-type rat showing no immunoreactivity against Aβ, using the human-specific anti-Aβ antibody McSA1. ***C***, Example slice from a 12-month-old homozygous transgenic rat with iAβ immunoreactivity. Insets show higher magnification of the subiculum (***D***, ***F***) and the MEC (***E***, ***G***). Scale bars: 500 µm (bar in ***A*** represents all overview images, bar in ***D*** all insets). CA3 and CA1, subfields of the hippocampus; Sub, subiculum; Per, perirhinal cortex.

In view of the minor increase in excitability observed in Layer II stellate cells in MEC, combined with the fact that these neurons provide major inputs to the DG (Cappaert et al., 2015), we decided to record the propagation of neural activity in the hippocampal region using VSDI in acute brain slices of McGill-R-Thy1-APP and wild-type rats (Fig. 1). Bipolar electrical stimulation was applied to the DG and MEC (Fig. 3*A*, areas shown with red asterisks). The slices used for VSDI were also immuno-stained for Aβ-42 and showed that in wild-type animals staining was absent (Fig. 3*B*,*D*,*E*), whereas every transgenic animal in all age groups (three, nine, and 12 months) had strong iAβ immunoreactivity in several areas of the hippocampal region (Fig. 3*C*,*F*,*G*). Expression was particularly strong in the pyramidal cell layer of subiculum (Fig. 3*F*), CA1, CA3 (Fig. 3*C*) as well as in Layer II of the EC (Fig. 3*G*). No extracellular plaques were seen in any of the slices from animals aged three or nine months, whereas at 12 months, the plaque levels were highly variable, from no plaques to very high plaque loads (Heggland et al., 2015).

### The neural network responses in the two blades of DG show subtle alterations in transgenic rats

**Figure 4.**
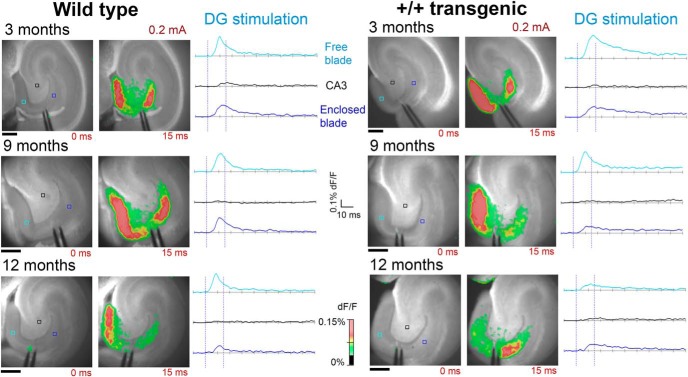
VSDI of DG showed differences in activation patterns in control and transgenic animals. Evoked activity from a bipolar stimulation electrode (single 0.2-mA pulse) centered in DG in control (left) and transgenic (right) animals. The activity spread to both blades, and in some cases, activity could also be seen in CA3. At three and nine months, the membrane potential changes were in general larger in the exposed blade than the enclosed blade. In contrast, at 12 months, several of the transgenic animals showed an increased activity in the inner blade. Images are from representative horizontal slices from wild-type and +/+ transgenic animals at three, nine, and 12 months of age. Scale bars: 500 µm.

Stimulation in the molecular layer in the crest of DG, the area bridging the two blades, with a single pulse (0.2 mA for 300 µs), resulted in activation in both of the blades of DG as well as in the hilus, and in several cases, a small change in the optical signal could also be seen in CA3 (Fig. 4). In the wild-type animals, the exposed blade (also called the outer, free, or infrapyramidal blade) had a higher level of activity than the enclosed blade (also called the inner or suprapyramidal blade), at all ages (Fig. 4, left panels). In the homozygous transgenic animals, this pattern of activation was also seen in the majority of the slices at three and nine months (Fig. 4, right panels). However, at 12 months of age, we observed that some transgenic rats had larger responses in the enclosed than the exposed blade or very similar responses in the two blades after stimulation in the crest (Fig. 4, lower right panel).

**Figure 5.**
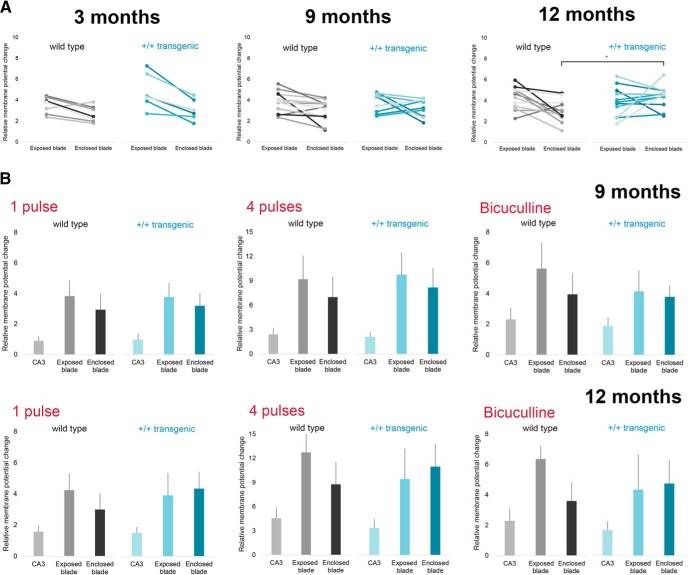
The evoked activity in the two blades of DG was significantly altered in 12-month-old transgenic animal, with increased activity in the enclosed blade, with a constant overall activated area. ***A***, Quantification of the relative membrane potential change measured with VSDI in the two blades of DG (ROI are the voxels shown in Fig. 3) is shown for three, nine, and 12 months. In control animals, the majority of the slices had larger membrane potential changes in the exposed blade than the enclosed blade, but at 12 months, this pattern was altered in a portion of the transgenic animals, while the total activated area was similar in all age groups. Values from singles slices are shown. Asterisk indicates estimated marginal means that have non-overlapping 95% confidence intervals. ***B***, Quantification of the relative membrane potential change measured with VSDI in the two blades of DG and CA3 in transgenic and control animals after a single or four-pulse stimulation and in the presence of 5 µM bicuculline at nine and 12 months. At nine months, the pattern of activation was similar in transgenic and control animals (top panels), but at 12 months, this pattern was altered in the +/+ transgenic group (bottom panels). Mean values are shown, with bar representing standard deviation. For each age group the effects of genotype and area and the interaction was tested with a linear mixed model. Results from the statistical analysis are shown in Extended Data Figure 5-1.

Quantification of the membrane potential changes were in line with this altered pattern of DG activation, following single pulse stimulation (Fig. 5; Extended Data Fig. 5-1). At three and nine months, there was a larger membrane potential change in the exposed blade than the enclosed blade in both wild-type and control animals (Fig. 5*A*, left and middle panel). The statistical analysis, using the mixed linear model, showed a significant effect of area (blade) at three and nine months, but no significant effect of genotype or interaction between area and genotype (Extended Data Fig. 5-1, rows a, b). At 12 months, the effect of area was no longer significant, nor was there a main effect of genotype (Extended Data Fig. 5-1, row c). However, at 12 months, there was a significant interaction between genotype and area (Extended Data Fig. 5-1, row c), and the membrane potential changes in the enclosed blade were significantly higher in the homozygous transgenic animals than in the wild-type animals (Fig. 5*A*, right panel). The same pattern of activation in DG was also found when stimulating with four pulses at 40 Hz, and with the addition of the GABAa antagonist bicuculline (Fig. 5*B*, middle and right panels). The statistical test showed comparable results, with a significant interaction between genotype and area at 12, but not nine, months (Extended Data Fig. 5-1, rows d–g).

Extended Data Figure 5-1 Results from the mixed linear model for quantified membrane potential change using VSDI in the DG of homozygous transgenic animals (+/+) and controls (–/–). Download Extended Data Figure 5-1, TIF file.

In all age groups, the total activated area (number of pixels) was unaltered in the +/+ transgenic rats compared to wild type (Table 5). In addition, there was no significant difference between the PPR in wild-type and +/+ transgenic animals at either nine or 12 months (Table 5). The addition of bicuculline did not change this pattern of activation. This indicates that although there was a change in the activation pattern of DG in the homozygous transgenic animals at 12 months, the overall excitability of the DG circuitry was not altered.

**Table 5.** Results from the mixed linear model for total number of activated pixels and PPR using VSDI in the DG of homozygous transgenic animals (+/+) and controls (–/–)

	Mean (SD)		*p* values for test of fixed effects		
	–/–	+/+	Genotype	Sex	*N* (slices/animals)
Activated pixels (1 pulse)					
3 months	807 (284)	83 (297)	0.549	0.463	13/7
9 months	1698 (579)	1800 (279)	0.641	0.638	21/10
12 months	1687 (485)	1581 (427)	0.564	0.733	23/9
PPR: normal ACSF					
9 months	0.860 (0.098)	0.811 (0.032)	0.157	0.198	21/10
12 months	0.920 (0.119)	0.854 (0.098)	0.190	0.718	23/9
PPR: bicuculline					
9 months	1.320 (0.076)	1.321 (0.082)	0.970	0.897	15/8
12 months	1.373 (0.088)	1.363 (0.070)	0.859	0.782	15/9

### Transient changes in neural network responses in MEC in transgenic rats

Layer II neurons in MEC that project to the hippocampus, also give rise to local axon collaterals in Layer I and II, reaching for around 300 µm with an occasional spread of up to 400 µm along the transverse axis (Tamamaki and Nojyo, 1993; Klink and Alonso, 1997; Schmidt et al., 2017). These local collaterals may innervate neurons in Layers II, III, and V, since all have apical dendrites in Layers I/II. We therefore aimed to look at differences in local network responses between wild-type and transgenic animals on stimulation in Layer II in MEC using VSDI.

Stimulation in superficial MEC (single 0.6-mA pulse) led to changes in the VSDI signal that spread in the superficial layers as well as to the deep layers of MEC (example image in Fig. 1*A*). In addition, small changes were observed in the pre- and parasubiculum and DG in many slices. We analyzed changes in fluorescent signal in individual voxels taken at the position of the stimulation electrode and gradually moving away with a maximum distance of ∼700 µm away from the electrode. As expected, a significant effect of distance from electrode was seen, with a decreasing signal with distance both across and within layers (Table 6; Extended Data Table 6-1). No main effect of genotype was observed for any age group (Table 6). However, at three months, there was a significant interaction between genotype and distance from electrode across layers, but this was not seen at nine and 12 months (Table 6). Similarly, a small but significant interaction between genotype and distance from electrode within layers was seen at nine months, but not three and 12 months (Table 6). These significant interactions indicate alterations in the network responses in the MEC of the transgenic rats. However, the effects are small and transient, as they are only seen in single age groups.

**Table 6.** Results from the mixed linear model for quantified membrane potential change using VSDI in MEC of homozygous transgenic animals (+/+) and controls (–/–)

	*p* values for test of fixed effects				
	Genotype	Sex	Distance from electrode	Distance from electrode × genotype	*N* (slices/animals)
Superficial layers					
3 months	0.095	0.116	**0.000**	0.652	16/6
9 months	0.315	0.431	**0.000**	**0.015**	21/10
12 months	0.445	0.297	**0.000**	0.696	13/7
Across layers					
3 months	0.237	0.266	**0.000**	**0.000**	16/6
9 months	0.639	0.104	**0.000**	0.902	21/10
12 months	0.673	0.750	**0.000**	1.000	13/7

Spread of activity from electrode placed in superficial layers MEC recorded with VSDI for all rats is shown in Extended Data Table 6-1. Values printed in bold face identify significance.

Extended Data Table 6-1 Spread of activity from electrode placed in superficial layers MEC recorded with VSDI in wild-type (wt) and transgenic (+/+) rats. The relative membrane potential change at increasing distance from the electrode tip is shown within the superficial layers (left) and across the layers of MEC (right), for three-, nine-, and 12-month-old rats. Download Extended Data T, TIF file.

## Discussion

Accumulating evidence suggests that the cognitive symptoms of memory loss and learning impairments in the early stages of AD are not mainly due to neuronal loss or atrophy, but can be linked to neuronal and synaptic dysfunction and subsequent abnormal patterns of activation in local neuronal circuits and larger-scale networks (D'Amelio and Rossini, 2012). Aβ peptides likely play an important role in these deleterious processes by affecting synapses and synaptic function (Palop and Mucke, 2010b). Here, we studied changes in the entorhinal-hippocampal network and single cells in acute slices taken from McGill-R-Thy1-APP transgenic rats expressing human mutated APP. We first analyzed the electrophysiological properties and excitability of the main principal cell populations in Layer II of EC, fan cells and stellate cells, with the use of *in vitro* whole-cell patch clamp. When comparing transgenic rats and controls, at one month and three to four months of age, we found no alterations in any of the passive membrane properties, and only subtle differences in the excitability of stellate cells. Further, with the use of VSDI, we observed alterations in the activation patterns of the two blades of DG in 12-month-old homozygous transgenic animals, as well as transient changes in the local network activity in MEC.

Other studies report network hyperexcitability, including seizures, in different brain areas of several mouse models of AD (Palop et al., 2007; Minkeviciene et al., 2009; Harris et al., 2010; Verret et al., 2012), including EC (Duffy et al., 2015; Xu et al., 2015) and DG (Hazra et al., 2013). In the current study, we did not find clear evidence for generalized hyperexcitability of EC or DG in the McGill rat using VSDI in slices. This apparent discrepancy likely is caused by the different experimental methods used. The optical signals we recorded represent the averaged membrane voltage changes in the total population of cells, including glial cells. An increased number of hyperactive as well as hypoactive neurons has previously been reported in AD mice using Ca^2+^ imaging (Busche et al., 2008, 2012). Such changes in single cells or ensembles would sum together and would therefore be difficult, if not impossible, to detect with VSDI. In addition, many of the studies reporting aberrant network activity in transgenic AD models have been done *in vivo*, with techniques including EEG (Minkeviciene et al., 2009; Verret et al., 2012), single neuron (Kellner et al., 2014), or local field potential recordings (Xu et al., 2015). Thus, we cannot exclude the possibility that *in vivo* recordings or using a different method to assess network function might reveal other changes in the McGill-R-Thy1-APP rat not seen in the present study.

We found no alterations of subthreshold intrinsic properties in either fan cell or stellate cells in the homozygous transgenic McGill rats aged one and three to four months. In Tg2576 mice, fan and stellate cells in EC showed no changes in the input resistance and resting membrane potential (Marcantoni et al., 2013), in agreement with our findings. Similar results with no changes in subthreshold properties have been shown in pyramidal cells in CA1 of the McGill rat (Qi et al., 2014), PSAPP (Brown et al., 2011), PDAPP (Kerrigan et al., 2014), 3xTg-AD (Scala et al., 2015), and CRND8 mice (Wykes et al., 2012) as well as in the frontal cortex of APPswe/PS1dE9 (APdE9) mice (Kellner et al., 2014). In contrast, a depolarization of the resting membrane potential has been found in interneurons in DG and pyramidal cells in neocortex in APdE9 mice (Minkeviciene et al., 2009; Hazra et al., 2013) in addition to parvalbumin-positive interneurons, but not pyramidal cells in parietal cortex of hAPPJ20 mice (Verret et al., 2012), suggesting that cell populations might be differentially affected.

We identified two suprathreshold properties that showed alterations in stellate cells in the homozygous rats. Stellate cells display a clear fAHP followed by a DAP (Alonso and Klink, 1993), and this fAHP is due to a Ca^2+^-dependent K^+^ conductance (Storm, 1987; Klink and Alonso, 1993). This conductance is thought to be mediated by BK (big potassium) channels, and is also important for spike repolarization (Sah, 1996). The BK channels can facilitate high-frequency firing, likely through limiting the activation of other potassium channels and decreasing the inactivation of sodium channels (Gu et al., 2007). Notably, the BK current is transient, inactivating rapidly, and thus will be most influential in the initial part of a spike train (Shao et al., 1999). Correspondingly, the other alteration we observed in stellate cells in the transgenic rats was increased excitability early in the spike train, a slightly higher *f_0_* at one month. It is thus possible that the hyperexcitability we here describe in the MEC stellate cells in the McGill rat actually results from early changes in ion conductances, in particular the BK potassium current, which might worsen over time. Whether the BK channel, or other channels, is affected specifically in this model will be of interest for further studies. Several studies report various physiologic alterations of single cells in other transgenic mouse models, including changes in excitability, potassium currents and AP wave form (Brown et al., 2011; Wykes et al., 2012; Kerrigan et al., 2014; Scala et al., 2015), highlighting several possible channels as targets for Aβ toxicity.

The VSDI data indicate an alteration of the response pattern in DG, seen in the 12-month homozygous transgenic group, but not at three and nine months. The McGill-R-Thy1-APP rat initially displays extracellular plaques at around nine months, and although the pattern of plaque deposition is similar across animals, the age of onset and temporal progression of the plaque pathology varied considerably between animals (Heggland et al., 2015). This corresponds to the findings in the current study, with highly variable levels of plaque in the recorded slices from the different homozygous transgenic animals. It is of interest to mention that one animal with the highest plaque load also had the largest change in DG activation pattern. In AD mice models, hyperactive neurons have been found to be associated with plaques (Busche et al., 2008, 2012) and deficits in place cell firing were related to hippocampal plaque burden in the Tg2576 model (Cacucci et al., 2008). Consistent with this are findings that synaptic density is reduced in proximity to plaques (Dong et al., 2007) and neurons in contact with plaques have a loss of perisomatic GABAergic synapses (Garcia-Marin et al., 2009), providing a possible mechanism for some of the observed network changes in AD models.

The observed responses after stimulation in the molecular layer of DG, which is a major area of termination of the perforant path input from Layer II of EC, revealed an asymmetry in the activation of the two blades in wild-type animals, with larger amplitudes in the exposed (infrapyramidal) blade than in the enclosed (suprapyramidal) blade. A similar asymmetry has been reported previously with VSDI in rats (Scharfman et al., 2002; Wright and Jackson, 2014) and Ca^2+^ imaging in mice (Yu et al., 2013). The inhibitory circuitry differs in the two blades (Seress and Pokorny, 1981), which could be a possible explanation for these observations. However, the asymmetry, as well as the alterations seen in the 12-month transgenic group, were seen using both normal ACSF and after addition of the GABA_A_ receptor antagonist bicuculline. This indicates that differences or alterations in inhibition do not play a major role in this case, although effects of GABA_B_ cannot be completely ruled out. Other known blade differences include the perforant path input from the EC, with the two blades receiving preferential input from different parts of EC (Wyss, 1981; Witter et al., 1989). Although the precise distribution of the perforant path to the two blades is somewhat disputed (Witter et al., 1989), these anatomic differences might play a role in the asymmetric activation of DG.

Although possible sex differences were not the focus of this study, the observed effects do support the inclusion of sex as a factor in future studies. In the statistical analysis of the electrophysiological parameters, we included age and sex as factors, both to be able to account for possible bias on the estimated effect of genotype, as well as the possibility that genotype had a differential effect on either sex or with increasing age. Women are at higher risk of developing AD (Li and Singh, 2014) and sex differences have been described in other animal models of AD (Wang et al., 2003). An effect of sex on changes in metabolism has been described in the McGill-R-Thy1-APP rat (Nilsen et al., 2014), although no clear differences were reported between males and females regarding plaque pathology (Heggland et al., 2015) or memory impairments (Leon et al., 2010). Some of the possible effects we find here might be due to different, and fluctuating, hormone levels, but also differences in genes and gene expression between sexes could play a part (Shah et al., 2014) The effects seen with age were also in general minor. Since we recorded in rats aged one month (juvenile) and three to four months (adult), the changes could reflect the transition to adulthood. Previously, stellate cells in MEC of young adult (postnatal day 46) rats have been shown to be less excitable, and have slight alterations in intrinsic electrophysiological properties, compared to juvenile (postnatal day 21) rats (Boehlen et al., 2010).

In summary, we found that in young animals, there were only minor alterations in the intrinsic electrophysiological parameters of single cells, with a slight hyperexcitability seen in stellate cells and no changes in the fan cells in the homozygous rats, although the majority of these cells displayed accumulation of iAβ. Following up on this, we found that the networks of DG and MEC were largely unaltered in the McGill-R-Thy1-APP transgenic rat. However, at 12 months, there was a statistically significant change in the typically asymmetric activation of the DG seen in wild-type rats. Additionally, there were transient changes in the local network of MEC. Whether the hyperexcitability of stellate cells plays a major role in the cognitive deficits seen in pre-plaque homozygous McGill rats still remains an open question. Additionally, the results from the VSDI point to the possible involvement of the medial perforant path to the DG in AD dysfunction. Even small alterations in the EC-DG or intrinsic DG circuitry could therefore perturb the normal hippocampal processing and thus affect learning and memory.

## Synthesis

Reviewing Editor: Jorge J. Palop, Gladstone Institutes and UCSF Decisions are customarily a result of the Reviewing Editor and the peer reviewers coming together and discussing their recommendations until a consensus is reached. When revisions are invited, a fact-based synthesis statement explaining their decision and outlining what is needed to prepare a revision will be listed below. The following reviewer(s) agreed to reveal their identity: Lori McMahon, Jonathan Brown.

This is comprehensive manuscript studying cellular and network dysfunction in the McGill-R-Thy1-APP transgenic AD rat using VSDI and whole-cell patch clamp recordings. However, during the discussion with the reviewers, we identified several critical aspects that need to be addressed (see the full reviews below):

1) The manuscript needs significant editing to present the information highlighting the critical findings (positive results) and condensing the negative results. The manuscript is difficult to read since the information is not presented efficiently (13 figures). For example, Figures 2, 3, 5 and 10 (1) only present single VSDI traces without mean values or statistics; (3) graphs contain no x- or y-labels; (2) single traces of transgenic and wildtype mice are in different graphs, which makes impossible to directly compare; (3) the figure legends for these figures just describe the raw traces without providing information about n numbers, means, or stats; (4) stats for these figures are independently presented in tables and described in different sections of the manuscript, which makes very hard to understand the significance of these results. We suggest to present means of wildtype and tg slices in the same graph with stats and n so a conclusion can be established in each figure.

2) Changes in network activity were only detected at 12 months of age, an age missing from the patch clamp recordings. The lack of these data certainly weakens the overall conclusion of the manuscript since the critical age is missing. Specific subheadings for the path-clamp data are needed (e.g., Figures 5, 6, 7, 8, and 9 all are under a subheading result). All these negative results (Figures 7, 8, 9, 11, 12, and 13) can be summarized in a table with the stats. The result section should focus on the significant effects (sex, age, and tg effects).

3) There are statements throughout the manuscript where the language is not clear and precise, creating technical and conceptual confusion (e.g, “appeared to show an effect of genotype”, “but these results should be interpreted with caution, as we did not adjust the significance level owing to multiple testing”). There are also some contradictions between the tables and the description, and the description and the raw data of the results that need to be resolved. The author proposed that early cognitive deficits at 3 months of age may be linked to alterations in intrinsic electrophysiological properties of neurons, which contradicts their own results.

4) Subtle deficits in voltage dependent dye response in the enclosed blade of the DG at 12 months. Clearly a lot of variability in Abeta expression in these rats - was there any correlation with levels of abeta pathology and functional network disruption? Can amyloid levels be included into the mixed model analyses?

Reviewer#1

This study investigates possible circuit dysfunction in the McGill-R-Thy1-APP transgenic AD rat using acute hippocampal brain slices in VSDI and whole-cell patch clamp recordings from stellate and fan cells in the entorhinal cortex. These functional studies have been complimented by immunohistochemical staining to locate accumulation of Abeta intracellularly and extracellularly in the form of plaques. The study is comprehensive in that 3 different ages (3,9, and 12 month old rats) from both sexes and from both genotypes were used in the VSDI studies, and 1 and 3-4 month old rats in the whole-cell studies. A tremendous amount of work has been done to determine whether synaptic dysfunction is occurring prior to the onset of significant Abeta plaque accumulation. While the authors found few significant differences between genotypes, even in the oldest age group (12 months), the findings are important to add to the literature for those using this preclinical model to understand human AD.

Major Comment: Overall, the study was well conducted, and based upon the description of the statistical analysis, it appears to be robust. The manuscript seems overly long, particularly in light of the mostly negative (although important) results. In many place the writing is much too dense, making it very difficult for the most important points to be extracted. There is too much emphasis describing findings that are not significantly different in both the results section as well as in the figure legends, which are also much too long. This comment also applies to the overly long discussion. The quality of the manuscript would be much improved if the manuscript undergoes significant editing.

Other Comments:

1) Despite the appearance of rigorous statistical analysis described in the methods section, there are statements throughout the manuscript where the language is not clear and precise. For example, in lines 268-270, what is meant by the statement, “...the stimulation in the molecular layer of DG was the only location that appeared to show an effect of genotype.”? If the differences do not reach statistical significance as indicated in the results section, then this statement is misleading. If there are differences, then say so and report the p value. If significant differences are not found, then say that directly too, and report the p value.

2) Along these same lines, in the discussion (approx. lines 741-746), the authors make note that the significant differences reported in the instantaneous frequency at the start of the spike train should be interpreted with caution, because it could be a false positive. This is a concern. In the tables, there are indications of significant differences in some parameters at some ages that are then lost at other ages. Are these significant differences important? Could they be false positives? Additional discussion is needed to help the reader know how to put these potential significant differences in context and to understand their meaning.

3) In lines 719-722 of the discussion, the authors refer to literature indicating that the McGill rat model displays hippocampus dependent learning and memory deficits as early as 3 months, and that this was correlated with levels of Abeta oligomers. They then state that interneuronal accumulation of Ab oligomers could paly a part in the observed dysfucntions by altering intrinsice electrophysiological properties of neurons, and thus cause aberrant activity in the affected neuronal network. This is confusing because the authors find no significant differences in intrinsic membrane properties. This needs editing for clarity.

4) In the methods, line 319-326, the authors describe exclusion criteria for the cells recorded in the whole-cell experiments. Given the different cell types in EC, why are the authors comfortable including the cells that were not successfully filled in the final analysis? This needs to be addressed and clarified.

5) Regarding immunohistochemical staining to visualized intracellular Ab, was a 4 day primary antibody incubation really needed? Please clarify. With the significant accumulation of intracellular Abeta, it seems surprising that there are no major functional differences found in the single cell recordings.

6) The figures could be better labeled for clarity, consistency, and with larger fonts for the axis labels.

Reviewer#2

This manuscript provides a thorough description of the electrical properties of the hippocampal formation in the ‘McGill’ transgenic rat which expresses a mutant form APP and produces some Abeta like pathology. The authors have used voltage sensitive dye imaging to assess the impact of electrical stimulation of the perforant path at a range of different ages, only observing differences at the most advanced age point test (12 months). The authors then go on to study the intrinsic membrane properties of LEC and MEC neurons at two relatively young age points (1 and 3-4 months). Here they found a subtle increase in excitability at the later age point of MEC stellate cells. The report is thorough and the experiments have been performed, analysed and presented to a high standard. On the whole, the authors did not find many differences between the genotypes, which may reflect the relatively weak pathological phenotype (i.e. Abeta deposition) observed in this model.

My only major concern is that whilst the changes in network activity were only detected at 12 months of age, the authors have not attempted to record the single cell properties at this age point. Ideally, the single cell properties would have been analysed at the 12 month age point as well - one might expect that the hyperexcitability phenotype becomes progressively more prominent, becoming sufficiently powerful to cause the network changes observed at this age point. The lack of these data certainly weakens the impact of the paper.

Other points:

1. There are a number of reports of variation in intrinsic properties of MEC stellate cells along the D-V axis (see the work by Giocomo and Nolan labs). I realise that the authors have confined their analysis 1.5 mm of the middle dorso-ventral layers, but it would be interesting to see if the authors observe a similar relationship as those previously report. Furthermore, there is evidence in a mouse model of tau pathology that these dorso-ventral gradients are disturbed (Booth et al 2016) - is there any evidence for a disruption to this functional anatomy in the Tg rats?

2. Subtle deficits in voltage dependent dye response in the enclosed blade of the DG at 12 months. Clearly a lot of variability in Abeta expression in these rats - was there any correlation with levels of abeta pathology and functional network disruption?

3. The authors have filled and reconstructed a fair proportion of their recorded neurons. Were there any changes to the morphology of these neurons in the Tg rat?

4. The fAHP here is measured as an absolute membrane value - what about the fAHP amplitude (i.e. difference between AP threshold and fAHP)?

5. The authors did not correct for liquid junction potential, but they should report what this is in the methods.
