# Supplementary figures and images for: Electrophysiological Characterization of Networks and Single Cells in the Hippocampal Region of a Transgenic Rat Model of Alzheimer’s Disease
Source: eNeuro. 2019 Feb 22;6(1):ENEURO.0448-17.2019. doi: 10.1523/ENEURO.0448-17.2019 (PMC6390198; doi:10.1523/ENEURO.0448-17.2019)

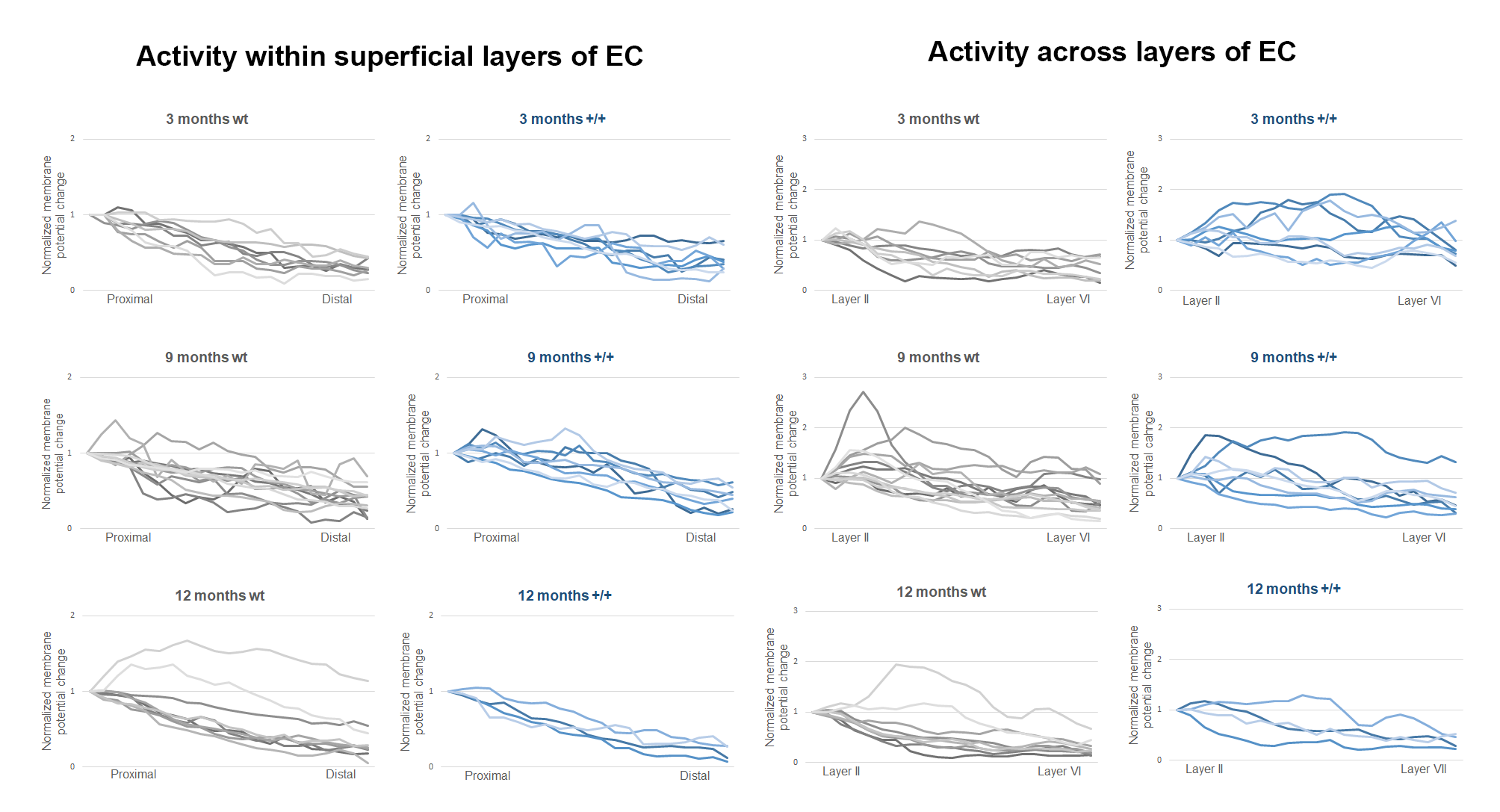

Supplement: Extended Data Table 1-1 — Basic electrophysiological and AP properties of fan cells in LEC LII in homozygous transgenic rats (+/+) and negative control animals (–/–) at one and three months of age. A, Input resistance measured from a series of current steps. The membrane time constant, τ (B); sag ratio (C); and rebound potential (D) all measured from a current step of –300 pA. E, Resting membrane potential, Vm. F, Rheobase, measured by current steps increasing by 10 pA/step. AP threshold (G), AP amplitude (H), and AP half width (I) all measured from the current step at rheobase. Values from all individual cells are shown (n = 111 cells in 40 animals). Download Extended Data Table 1-1, TIF file. [file sup_enu-eN-NWR-0448-17-s06.tif]

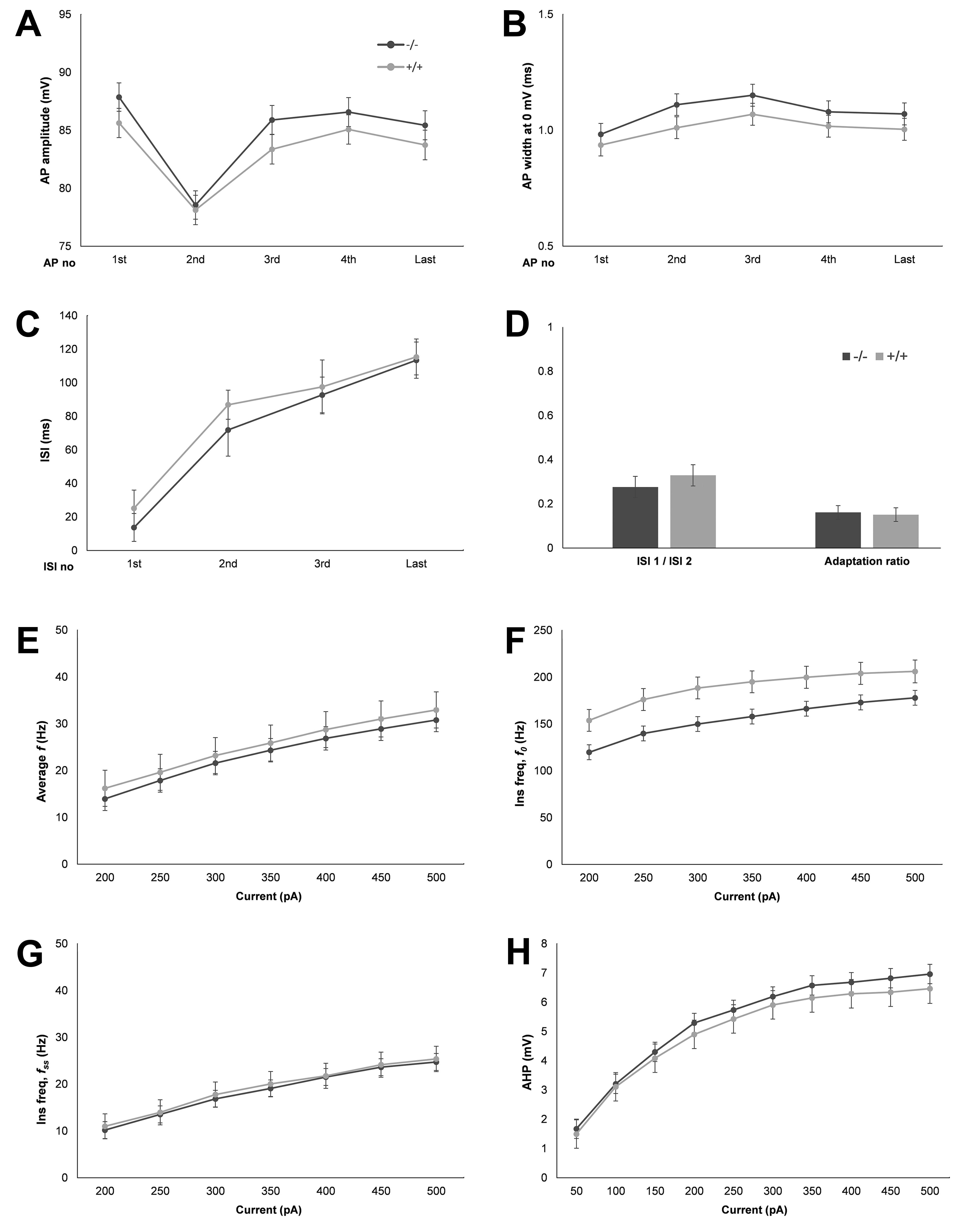

Supplement: Extended Data Table 3-1 — Basic electrophysiological properties of MEC LII stellate cells in homozygous transgenic rats (+/+) and negative control animals (–/–) at one and three months of age. A, Input resistance measured from a series of current steps. The membrane time constant, τ (B); sag ratio (C); and rebound potential (D), all measured from a current step of –300 pA. E, Resting membrane potential, Vm. F, Membrane resonance frequency in response to a ZAP current. G, Rheobase, measured by current steps increasing by 10 pA/step. AP threshold (H), AP amplitude (I), AP half width (J), fAHP, fAHP (K), and DAP (L), all measured from the first AP of the current step at rheobase. Values from all individual cells are shown (n = 78 cells in 30 animals). Download Extended Data Table 3-1, TIF file. [file sup_enu-eN-NWR-0448-17-s04.tif]

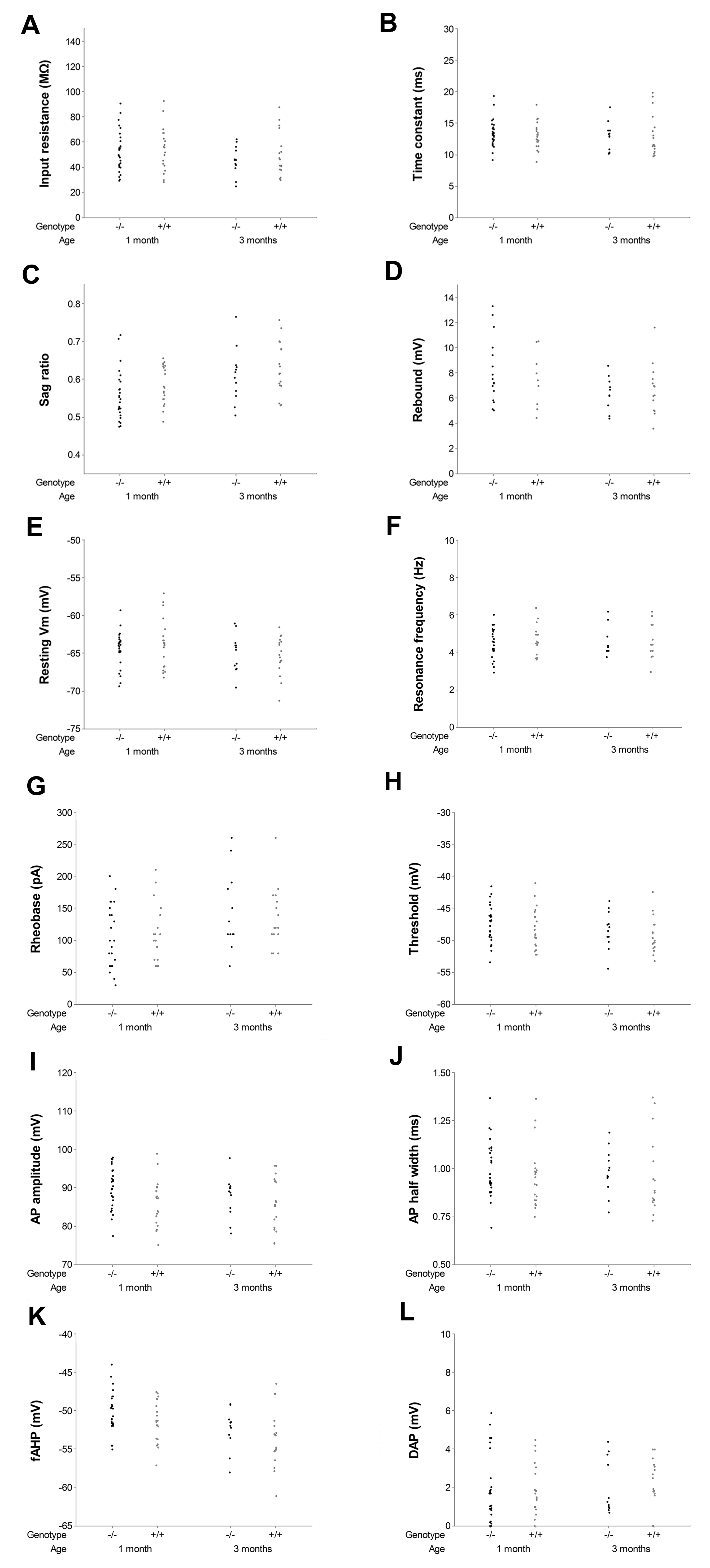

Supplement: Extended Data Table 4-1 — AP and firing properties of MEC LII stellate cells in homozygous transgenic rats (+/+) and control animals (–/–), for both age groups in A–D, one-month group in E–H. A, AP amplitude as a function of AP number. B, AP width at 0 mV as function of AP number. C, ISI, interspike interval as a function of spike interval number. D, Ratio of the two first interspike intervals (ISI1/ISI2) and adaptation ratio (first ISI/last ISI). Values in A–D are measured from a +200-pA current step (n = 78 cells in 30 animals). Average firing frequency, f (E); instantaneous firing frequency between two first spikes, f0 (F); instantaneous firing frequency between two last spikes, fss (G); and afterhyperpolarizing potential after end of current step (H), all plotted as a function of current (n = 38 cells in 16 animals). All values are shown as estimated marginal means and SEs from the mixed linear model. Download Extended Data T, TIF file. [file sup_enu-eN-NWR-0448-17-s03.tif]

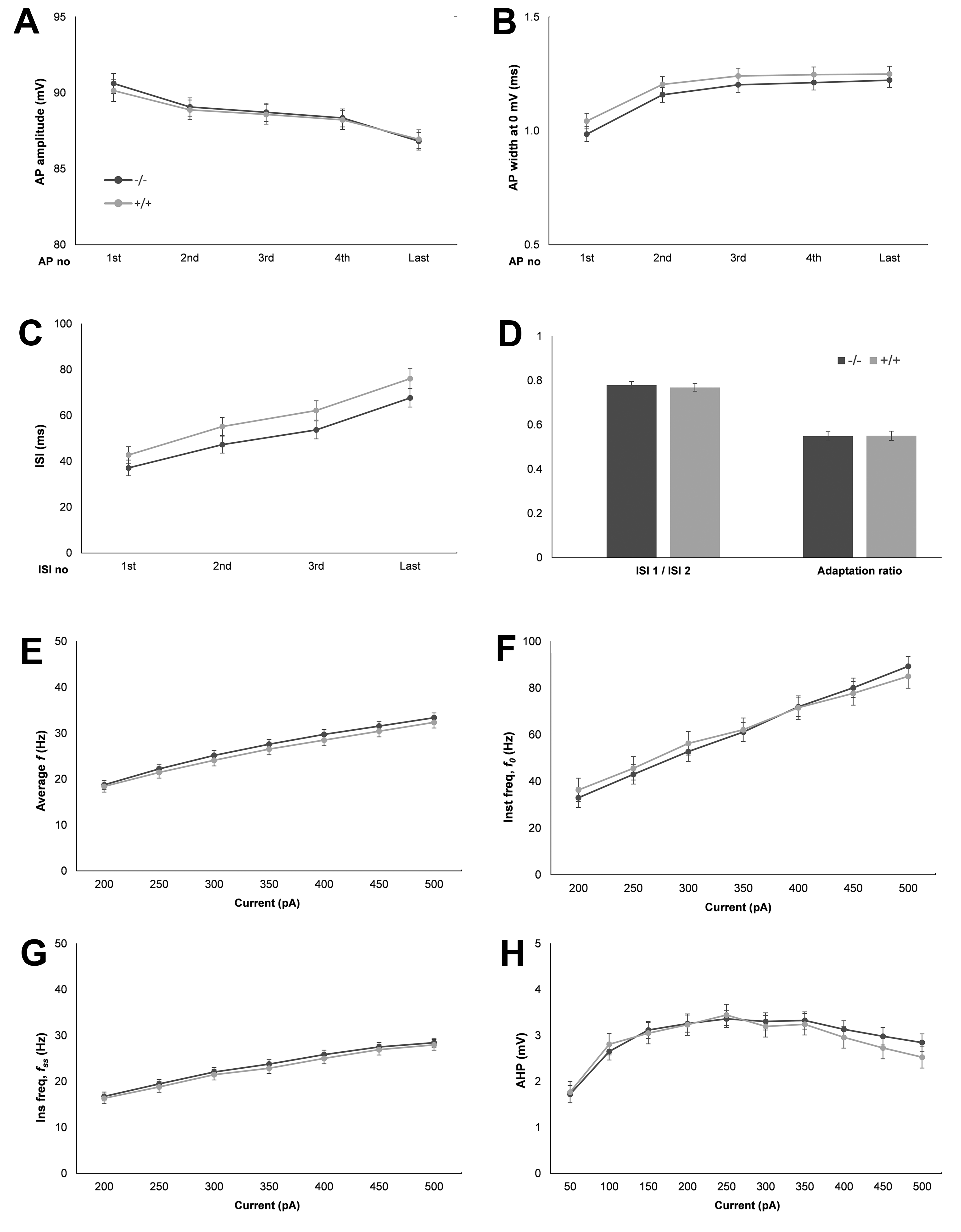

Supplement: Extended Data Figure 5-1 — Results from the mixed linear model for quantified membrane potential change using VSDI in the DG of homozygous transgenic animals (+/+) and controls (–/–). Download Extended Data Figure 5-1, TIF file. [file sup_enu-eN-NWR-0448-17-s02.tif]

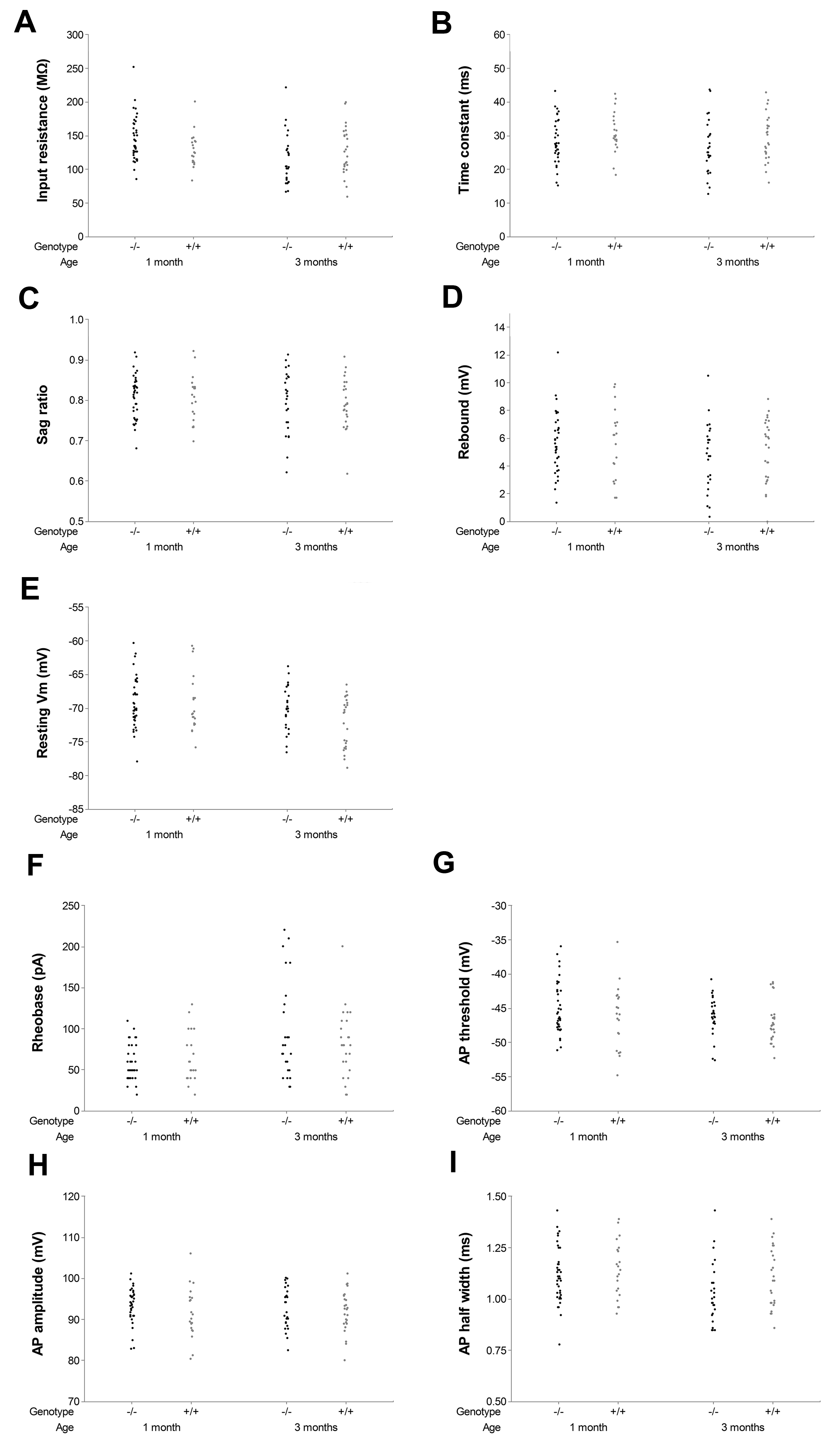

Supplement: Extended Data Table 6-1 — Spread of activity from electrode placed in superficial layers MEC recorded with VSDI in wild-type (wt) and transgenic (+/+) rats. The relative membrane potential change at increasing distance from the electrode tip is shown within the superficial layers (left) and across the layers of MEC (right), for three-, nine-, and 12-month-old rats. Download Extended Data T, TIF file. [file sup_enu-eN-NWR-0448-17-s01.tif]
