# Supplementary material for: Electrophysiological Characterization of Networks and Single Cells in the Hippocampal Region of a Transgenic Rat Model of Alzheimer’s Disease
Source: eNeuro. 2019 Feb 22;6(1):ENEURO.0448-17.2019. doi: 10.1523/ENEURO.0448-17.2019 (PMC6390198; doi:10.1523/ENEURO.0448-17.2019)
Supplement: Extended Data Table 2-1 — AP and firing properties of LEC LII fan cells in homozygous transgenic rats (+/+) and control animals (–/–), in both age groups. A, AP amplitude as a function of AP number. B, AP width at 0 mV as function of AP number. C, ISI, interspike interval as a function of spike interval number. D, Ratio of the two first interspike intervals (ISI1/ISI2) and adaptation ratio (first ISI/last ISI). Values in A–D are measured from a +200- or 210-pA current (n = 111 cells in 40 animals). Average firing frequency, f (E); instantaneous firing frequency between two first spikes, f0 (F); instantaneous firing frequency between two last spikes, fss (G); afterhyperpolarizing potential after end of current step (H), all plotted as a function of current (n = 67 cells in 26 animals). All values are shown as estimated marginal means and SEs from the mixed linear model. Download Extended Data Table 2-1, DOCX file. [file sup_enu-eN-NWR-0448-17-s05.docx]

**Figure 5.1.** Results from the mixed linear model for quantified membrane potential change using VSDI in the DG of homozygous transgenic animals (+/+) and controls (-/-).

|  |  | **p-values for test of fixed effects** | | | |  |
| --- | --- | --- | --- | --- | --- | --- |
|  |  | **Genotype** | **Area** | **Genotype x Area** | **Sex** | **N**  **(slices/animals)** |
|  | **1 pulse** |  |  |  |  |  |
| a | 3 months | 0.177 | **0.008** | 0.316 | 0.753 | 13/7 |
| b | 9 months | 0.757 | **0.014** | 0.583 | **0.022** | **21/10** |
| c | 12 months | 0.191 | 0.243 | **0.019** | 0.192 | 23/9 |
|  | **4 pulses** |  |  |  |  |  |
| d | 9 months | 0.28 | **0.017** | 0.68 | **0.005** | **21/10** |
| e | 12 months | 0.394 | 0.208 | **0.007** | 0.169 | 23/9 |
|  | **Bicuculline** |  |  |  |  |  |
| f | 9 months | 0.155 | 0.069 | 0.197 | 0.496 | 15/8 |
| g | 12 months | 0.596 | 0.066 | **0.016** | 0.596 | 15/9 |
